# Exosome-encapsulated microRNAs as circulating biomarkers for colorectal cancer

**DOI:** 10.18632/oncotarget.18557

**Published:** 2017-06-16

**Authors:** Shushan Yan, Bing Han, Shunyuan Gao, Xiaochen Wang, Zengfang Wang, Fakai Wang, Jianjun Zhang, Donghua Xu, Beicheng Sun

**Affiliations:** ^1^ Key Laboratory on Living Donor Liver Transplantation, Ministry of Health, Department of Liver Surgery, Collaborative Innovation Center For Cancer Personalized Medicine, First Affiliated Hospital of Nanjing Medical University, Nanjing, China; ^2^ Department of Gastrointestinal and Anal Diseases Surgery, The Affiliated Hospital of Weifang Medical University, Weifang, China; ^3^ Department of Clinical Laboratory, People's Hospital of Zoucheng, Zoucheng, China; ^4^ Department of Neurology, The Affiliated Huai'an Hospital of Xuzhou Medical University and The Second People's Hospital of Huai'an, Huai'an, China; ^5^ Department of Gynecology and Obstetrics, Weifang Hospital of Maternal and Child Health, Weifang, China; ^6^ Department of Neurosurgery, The Affiliated Hospital of Weifang Medical University, Weifang, China; ^7^ Department of Obstetrics, The Affiliated Hospital of Weifang Medical University, Weifang, China; ^8^ Department of Rheumatology and Immunology, The Affiliated Hospital of Weifang Medical University, Weifang, China; ^9^ Clinical Medicine College, Weifang Medical University, Weifang, China

**Keywords:** exosomes, microRNAs, colorectal cancer

## Abstract

Currently available studies have suggested that a number of exosome-encapsulated microRNAs (miRNAs) are recognized as stable biomarkers for cancers. However, little is known about the effect of exosomal miRNAs on colorectal cancer (CRC). The aim of study is to identify specific miRNAs in serum exosomes, which may serve as potential diagnostic and prognostic biomarkers and therapeutic targets for CRC. Microarray analyses of miRNAs in serum exosomes from 3 primary CRC patients and 3 healthy controls were performed. Those differentially expressed exosome-encapsulated miRNAs were verified in exosome-enriched serum samples from 77 CRC patients and 20 healthy controls by quantitative real-time PCR (qRT-PCR). A total of 39 aberrantly expressed miRNAs in serum exosomes were identified by microarray analysis. After confirmation by qRT-PCR, we found that 5 exosome-encapsulated miRNAs (miR-638, miR-5787, miR-8075, miR-6869-5p and miR-548c-5p) were significantly down-regulated, while 2 exosome-encapsulated miRNAs (miR-486-5p and miR-3180-5p) were significantly up-regulated in serum. Decreased levels of miR-638 in serum exosomes were associated with increased risk of liver metastasis and later TNM stage of CRC. Networks analyses revealed that 5 aberrantly expressed miRNAs (miR-638, miR-5787, miR-8075, miR-6869-5p, and miR-548c-5p) might be involved in the process of glucose metabolism in CRC. The present study shows the specific serum profile of exosome-encapsulated miRNAs in CRC. Those specific miRNAs in serum exosomes may serve as disease biomarkers and novel therapeutic targets for CRC.

## INTRODUCTION

Colorectal cancer (CRC) is a common digestive cancer and one of the major causes of cancer-related deaths worldwide [1, 2]. Although great progress has been made in the early diagnosis and treatment of CRC, the societal and economic burdens of CRC will significantly worsen over the coming decades in that the number of young adults with CRC is growing [3, 4]. Given early screening, early diagnosis and early treatment are essential for individuals at risk for CRC, identifying valuable circulating or tissular biomarkers merits investigation. To the best of our knowledge, carcinoembryonic antigen (CEA) and carbohydrate antigen 19-9 (CA19-9) are well-established tumor markers for the detection of many types of digestive cancer, including CRC [5, 6]. There is insufficient evidence for the routine use of certain factors, such as p53, K-ras and deleted in colon cancer (DCC), as biomarkers either for estimating the prognosis or predicting response to chemotherapy in patients with CRC [6]. Moreover, lack of specificity and sensitivity preclude the use of such biomarkers for the early detection of CRC, especially for those individuals at an early stage of the disease. Thus, identifying novel CRC-specific biomarkers with high sensitivity is of great promise for the early diagnosis and prognosis estimation of patients with CRC.

Accumulating evidence has suggested that aberrantly expressed circulating noncoding RNAs, such as microRNAs (miRNAs), long noncoding RNAs (lncRNAs) and circular RNAs, can regulate the complicated process of cell proliferation, apoptosis and angiogenesis, and thus contribute to tumorigenesis and tumor progression [7–10]. Accordingly, these noncoding RNAs may be valuable noninvasive tools that can be used as diagnostic and prognostic biomarkers and therapeutic targets for cancer. MiRNAs are small non-coding RNAs with 18–25 nucleotides in length, which regulate gene expression at the transcriptional level and various crucial cell processes such as cell proliferation, apoptosis, differentiation and development [11, 12]. Abnormal expression profiles of miRNAs are related to a variety of tumors, including CRC. Specific miRNAs can act as either tumor suppressors or oncogenes. Increasing studies have shown aberrantly expression profiles of microRNAs in CRC, which are associated with the diagnosis, prognosis, and therapeutic outcome of patients with CRC [11, 13, 14]. In addition, miRNAs are also involved in intercellular signal transduction mediated by exosomes [15, 16]. It has been well documented that miRNAs can be released from cancer cells into body fluids via exosomes, including serum, milk, urine, and saliva, while exosomes are 40–100 nm diameter membrane vesicles that embed protein, lipids, mRNAs, and miRNAs [16, 17]. The contents are different depending on the origin of the secreting cells [16, 17]. Inappropriate release or dysregulation of exosomal miRNAs may lead to significant alterations in certain biological processes that influence cancer development and progression. Therefore, exosome-encapsulated miRNAs may serve as potential diagnostic and prognostic biomarkers and therapeutic targets for cancer. However, little is known about the association between exosomal miRNAs and CRC. As a result, we performed this microarray-based profiling of exosomal miRNAs in serum from CRC patients to demonstrate the underlying molecular networks involved in CRC development and progression, which may serve as disease biomarkers and novel therapeutic targets.

## RESULTS

### Specific miRNAs expression profile in serum exosomes from patients with CRC

**Figure 1:**
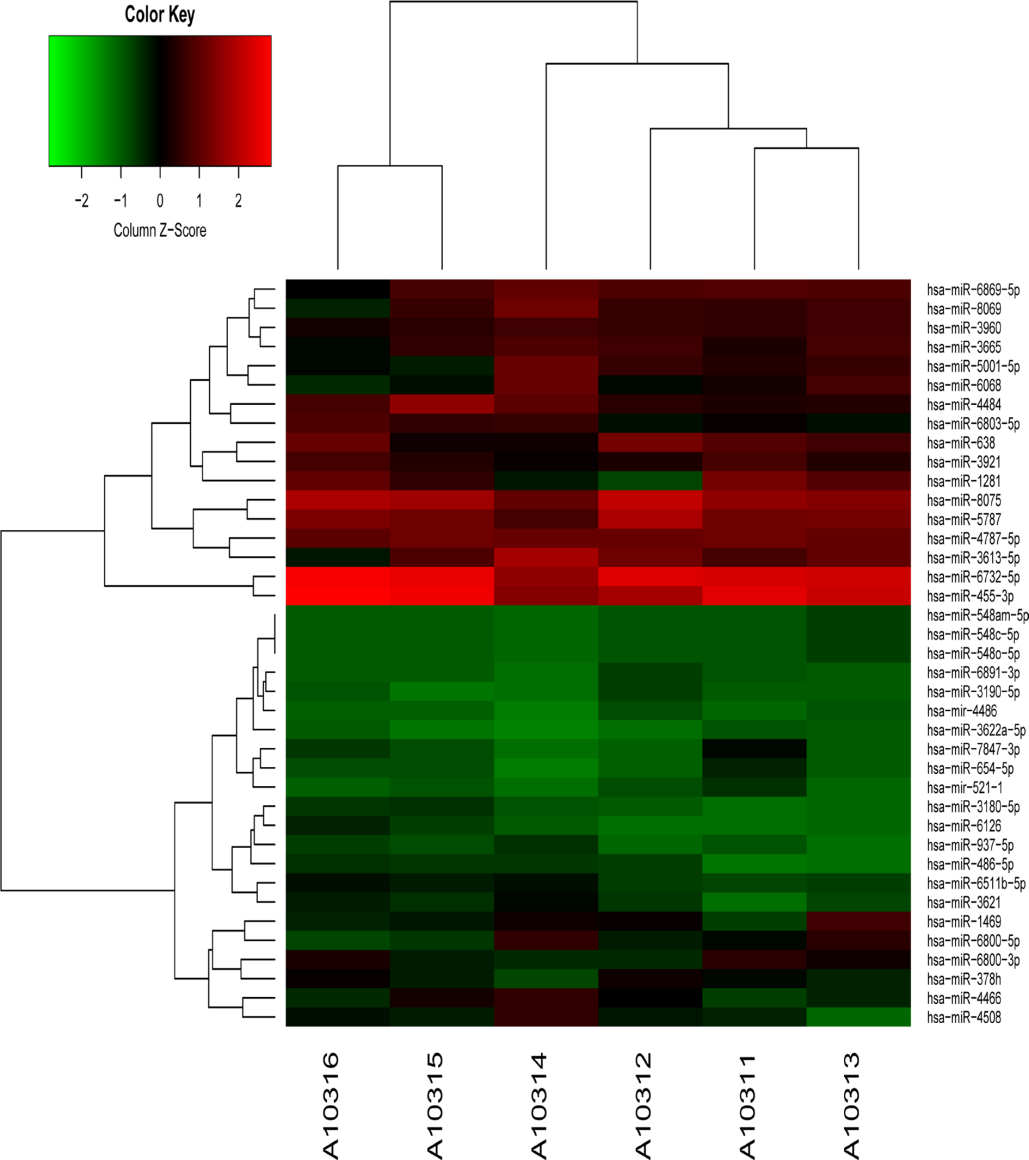
Hierarchical clustering analysis for specific miRNAs expression profile (39 aberrantly expressed exosomal miRNAs) in serum exosomes of patients with CRC (CRC group: A10314, A10315, A10316; Control group: A10311, A10312, A10313).

**Figure 2:**
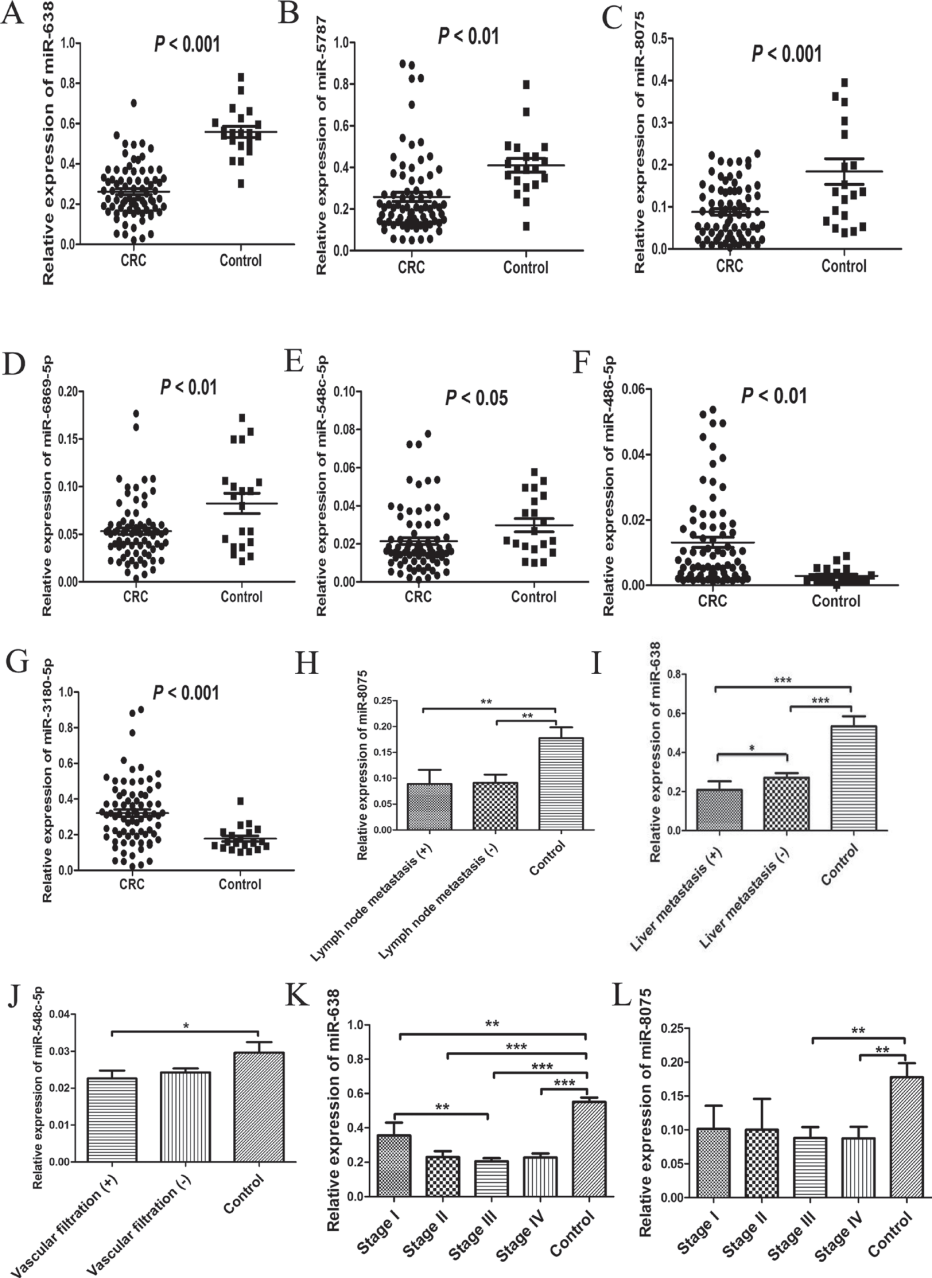
Validation of differentially expressed serous exosomal miRNAs and their association with characteristics of CRC patients (**A)** Relative expression of miR-638 in serum exosomes from 77 CRC patients and 20 healthy controls; (**B)** Relative expression of miR-5787 in serum exosomes from 77 CRC patients and 20 healthy controls; (**C)** Relative expression of miR-8075 in serum exosomes from 77 CRC patients and 20 healthy controls; (**D)** Relative expression of miR-6869-5p in serum exosomes from 77 CRC patients and 20 healthy controls; (**E)** Relative expression of miR-548c-5p in serum exosomes from 77 CRC patients and 20 healthy controls; (**F)** Relative expression of miR-486-5p in serum exosomes from 77 CRC patients and 20 healthy controls; (**G)** Relative expression of miR-3180-5p in serum exosomes from 77 CRC patients and 20 healthy controls; (**H)** Association between the relative expression of miR-8075 in serum exosomes and lymphatic infiltration of CRC (**P < 0.01); (**I**) Association between the relative expression of miR-638 in serum exosomes and liver metastasis of CRC (***P < 0.001); (**J)** Association between the relative expression of miR-548c-5p in serum exosomes and vascular filtration of CRC (*P < 0.05); (**K)** Association between the relative expression of miR-638 in serum exosomes and TNM stage of CRC (**P < 0.01; ***P < 0.001); (**L**) Association between the relative expression of miR-8075 in serum exosomes and TNM stage of CRC (**P < 0.01).

There were a total of 39 aberrantly expressed miRNAs in serum exosomes from patients with CRC were identified by microarray analysis. Among them, 10 miRNAs were up-regulated, while 29 miRNAs were down-regulated (Figure 1 and Table 1). After confirmation by quantitative real-time PCR (qRT-PCR), we found that 5 exosome-encapsulated miRNAs including miR-638, miR-5787, miR-8075, miR-6869-5p, and miR-548c-5p were significantly down-regulated, while 2 exosome-encapsulated miRNAs including miR-486-5p and miR-3180-5p were significantly up-regulated in serum exosomes of CRC patients (Figure 2A–2G).

**Table 1:** Specific miRNAs expression profile in serum exosomes of patients with CRC

No.	MiRNAs	Absolute fold change	Regulation
1	miR-638	2.930560592	down
2	miR-5787	2.679362906	down
3	miR-8075	2.181857177	down
4	miR-6732-5p	2.032971514	down
5	miR-6803-5p	1.96943709	up
6	miR-6869-5p	1.948076051	down
7	miR-4508	1.931613788	up
8	miR-4484	1.845783488	up
9	miR-5001-5p	1.833097259	down
10	miR-6800-5p	1.775103731	down
11	miR-6800-3p	1.761999278	down
12	miR-378h	1.761991136	down
13	miR-3960	1.728455347	down
14	miR-3613-5p	1.726623265	down
15	miR-3621	1.724765629	up
16	miR-6126	1.680524606	up
17	miR-486-5p	1.639642549	up
18	miR-455-3p	1.634149529	down
19	miR-3921	1.594429159	down
20	miR-3665	1.583818115	down
21	miR-548c-5p	1.570343313	down
22	miR-548am-5p	1.570343313	down
23	miR-548o-5p	1.570343313	down
24	miR-4787-5p	1.564856103	down
25	miR-1469	1.558725265	down
26	miR-4466	1.554851358	up
27	miR-1281	1.549704701	down
28	miR-8069	1.545496085	down
29	miR-6511b-5p	1.529489285	up
30	miR-6891-3p	1.527919286	down
31	mir-521-1	1.521360796	down
32	miR-7847-3p	1.513805653	down
33	miR-3622a-5p	1.513540556	down
34	miR-3180-5p	1.512824885	up
35	miR-654-5p	1.511857677	down
36	mir-4486	1.511099857	down
37	miR-937-5p	1.509192323	up
38	miR-3190-5p	1.50628455	down
39	miR-6068	1.5043393	down

### Relationship between serum exosomal miRNAs and the progression of CRC

Low levels of miR-638 in serum exosomes were associated with increased risk of liver metastasis and later TNM stage of patients with CRC (Figure 2I and 2K). However, there was no significant association regarding other parameters including lymphatic infiltration and vascular filtration (Figure 2H, 2J and 2L).

### MiRNAs may influence the glucose metabolism in CRC

**Figure 3:**
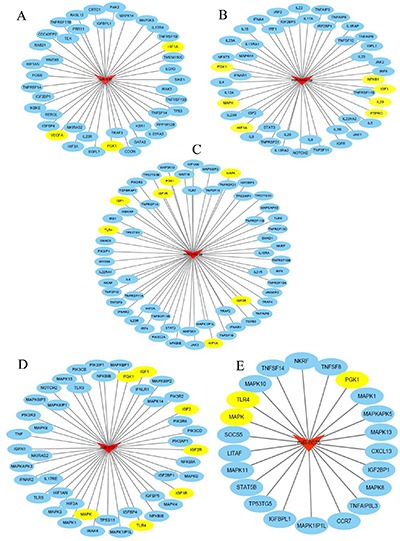
Bioinformatics analysis for potential targeted genes (**A)** The targeted genes of miR-638; (**B)** The targeted genes of miR-548c-5p; (**C)** The targeted genes of miR-6869-5p; (**D)** The targeted genes of miR-5787; (**E)** The targeted genes of miR-8075. (The pictures were created by use of Cytoscape software (version 3.4.0). Red: exosomal miRNAs; Blue: potential targeted genes; Yellow: potential targeted genes involved in the regulation of glucose metabolism).

Potential targeted genes of miRNAs were predicted in databases of targetscan (http://www.targetscan.org/vert_71/), microRNA.ORG (http://www.microrna.org/microrna/home.do) and miRDBA (http://www.mirdb.org/miRDB/). Networks analyses showed that 5 aberrantly expressed miRNAs (miR-638, miR-5787, miR-8075, miR-6869-5p and miR-548c-5p) might be involved in the process of glucose metabolism in CRC (Figure 3).

## DISCUSSION

The present study presents the specific serum profile of exosome-encapsulated miRNAs in CRC. There are 39 differentially expressed miRNAs in serum exosomes detected by microarray analysis. After validation by qRT-PCR, 5 exosome-encapsulated miRNAs including miR-638, miR-5787, miR-8075, miR-6869-5p and miR-548c-5p are observed to be significantly down-regulated, while 2 exosome-encapsulated miRNAs including miR-486-5p and miR-3180-5p are significantly up-regulated in serum samples from patients with CRC. In addition, miRNAs of miR-638, miR-5787, miR-8075, miR-6869-5p and miR-548c-5p may targetedly regulate the metabolism of glucose, thus playing critical roles in tumorigenesis and tumor progression. Those aberrantly expressed miRNAs in serum exosomes may serve as CRC biomarkers and novel promising therapeutic targets. Nonetheless, the underlying molecular mechanisms of those specific exosome-encapsulated miRNAs warrant further investigation.

Screening by use of colonoscopy in adults aged more than 50 years old makes the rate of CRC early detection much higher than before. However, the compliance with electronic colonoscopy screening is poor. Besides, the specificity and sensitivity of common tumors markers for CRC is not good enough, such as CEA and CA19-9 [5, 6]. Accordingly, finding a non-invasive and blood-based test for early detection seems essential. Circulating non-coding RNAs have increasingly emerged as new biomarkers for cancers, such as miRNAs, lncRNAs and circular RNAs. Accumulating data have suggested that these non-coding RNAs may exert crucial regulatory effects on cancer development, progression and metastasis [18–20]. It has been well documented that miRNAs belongs to the non-coding RNAs, which can act as either tumor suppressors or oncogenes [21]. Non-coding miRNAs stably exist in body fluids, such as plasma and exosomes, and can modulate multiple target genes at the post-transcriptional level. It has been well documented that miRNAs are vital cogs in numerous biological processes. Dysregulation and aberrant expression of miRNAs are often observed in a variety of tumors, which leads to immune disorders or immune evasion and induces tumorigenesis. Some anti-miRNAs can be used as effective anticancer therapies by targeting certain oncogenes such as epidermal growth factor receptor (EGFR) and human epidermal growth factor receptor 2 (HER2) [22]. A recent study has suggested that miRNA could inhibit cell proliferation and induce apoptosis of cancer cells by targeting sphingosine kinase 2 (SphK2), a factor promoting tumor progression [23]. Thus, miRNAs may also influence the progression of malignant tumors. Previous studies have implicated that a number of miRNAs are involved in colorectal carcinogenesis and cancer progression, such as miR-27a and miR-484 [21, 24–26], which can be used as disease markers for diagnosis and/or prognosis or therapeutic targets for CRC.

Besides CRC carcinogenesis, miRNAs are also demonstrated to participate in the regulation of other biological processes, such as development, proliferation, apoptosis, angiogenesis, migration, and metastasis [25]. In recent years, exosome-encapsulated microRNAs have emerged as a new class of biomarkers for diagnosis and prognosis evaluation of CRC [27, 28]. Zhang J and the colleagues have found that miR-638 was down-regulated and functioned as a tumor suppressor by inhibiting TSPAN1 in human CRC [29]. Similarly, reduced level of miR-638 is observed in exosomes from serum samples of patients with CRC in our study. Besides, there are still 4 miRNAs significantly down-regulated in serum exosomes of CRC patients compared with healthy controls, namely miR-5787, miR-8075, miR-6869-5p and miR-548c-5p. Moreover, the two exosome-encapsulated miRNAs of miR-486-5p and miR-3180-5p are found to be significantly up-regulated in serum exosomes of CRC patients in contrast to healthy controls. On the contrary, Liu C et al. have reported that miR-486-5p could act as a tumor-suppressor miRNA in CRC [30]. They found that miR-486-5p was significantly downregulated in CRC tissues compared with paracancer tissues [30], suggesting miR-486-5p might be a suppressor of CRC. This discrepancy may be attributed to different source of miRNAs, region of origins and sample size. More future studies are warranted to clarify this issue and still in further regard to miR-8075 and miR-548-5c with unknown targets and cellular function so far. Taken together, these aberrantly expressed miRNAs may be potential disease markers and therapeutic targets for CRC. Nevertheless, the precise roles and regulatory effects of these exosome-encapsulated miRNAs warrant to be elucidated in more future studies with high quality.

It has been demonstrated that miRNAs may influence the progression and prognosis of CRC patients. MiR-21 is found to be highly expressed in many types of cancer including CRC, and it is one of the most investigated miRNAs as a circulating prognostic biomarker for CRC [31, 32]. Some miRNAs are identified as predictors of lymphatic infiltration or vascular infiltration or recurrence of CRC, such as miR-17-92a and miR-92a [32–34]. In our study, low levels of exosomal miR-638 in serum exosomes were found to be associated with increased risk of liver metastasis. Additionally, CRC patients with low levels of miR-638 in serum exosomes were usually at later TNM stage. Taken together, circulating exosomal miRNAs are promising prognostic markers for CRC, which helps to guide the treatment for CRC patients at different course.

During the past decade, the role of non-coding RNAs in cancer metabolism has drawn much attention [20]. MiRNAs have emerged as an important kind of molecule that can regulate altered genes involved in metabolic reprogramming, while metabolic reprogramming is an important hallmark of cancer [35]. Increasing evidence has suggested critical roles of exosome-delivered miRNAs in metabolic reprogramming and cancer cells communication [36, 37]. Exosome-delivered miRNAs may regulate tumor angiogenesis, metastasis and immune escape by interacting with stromal cells in the tumor microenvironment. A previous study showed that miR-34a, miR-34c, miR-369-3p, miR-374a, and miR-4524a/b can targetedly regulate Lactate dehydrogenase A and influence the glycolysis in CRC [38], suggesting an important role of miRNAs in cancer glucose metabolism. Similar findings were demonstrated by Li X, et al. [39]. It has been shown that miR-106a could significantly reduce the expression of hypoxia-inducible factor-1α (HIF-1α) and vascular endothelial growth factor (VEGF), which could prevent high glucose-induced increased permeability. All these findings reveal that miRNAs are involved in the process of glucose metabolism and influence colorectal carcinogenesis and progression. The network analyses in the current study also implicate miRNAs of miR-638, miR-5787, miR-8075, miR-6869-5p and miR-548c-5p are aberrantly expressed in the serum exosomes of patients with CRC, and these miRNAs might modulate the glucose metabolism in CRC by targeting HIF-1α, VEGF, PGK1, MAPK and other molecules involved in glucose metabolism. However, more research is warranted to elucidate the precise molecular mechanisms with regard to miRNAs and glucose metabolism in CRC.

In summary, our study shows the specific miRNAs expression profile in serum exosomes of patients with CRC. Five aberrantly expressed miRNAs (miR-638, miR-5787, miR-8075, miR-6869-5p and miR-548c-5p) might influence the development and progression of CRC by regulating the glucose metabolism of cancer cells. Those specific miRNAs in serum exosomes may serve as disease biomarkers and novel therapeutic targets for CRC. However, collaborative and multi-center large retrospective and/or prospective cohort studies are warranted for further investigation.

## MATERIALS AND METHODS

### Patients and sample collection

77 CRC patients were recruited from the affiliated hospital of Weifang Medical University between March, 2014 and January, 2017. Healthy controls were recruited from the same hospital for health examination. The cell-free serum samples were collected by sequentially centrifuging fresh blood samples at 2000 rpm for 10 min. and stored at −80°C for further detection. Table 2 showed characteristics of all CRC patients and healthy controls.

**Table 2:** Characteristics of all CRC patients and healthy controls

Factors	CRC (*n* = 77)	Healthy controls (*n* = 20)
Age, *n* (%)		
< 58 years	38 (49.4)	9 (45.0)
≥ 58 years	39 (50.6)	11 (55.0)
Gender, *n* (%)		
Female	36 (40.6)	8 (40.0)
Male	41 (59.4)	12 (60.0)
TNM stage, *n* (%)		
I	15 (19.5)	
II	11 (14.3)	
III	33 (42.8)	
IV	18 (23.4)	
Tumor differentiation status, *n* (%)		
High	49 (63.6)	
Low	28 (36.4)	
Lymphatic infiltration, *n* (%)		
Yes	20 (26.0)	
No	57 (74.0)	
Liver metastasis, *n* (%)		
Yes	12 (15.6)	
No	65 (84.4)	
Vascular filtration, *n* (%)		
Yes	37 (48.1)	
No	40 (51.9)	

### Isolation of exosomes from serum

Exosomes from serum samples of 77 patients and 20 controls were isolated using a Total Exosome Isolation Kit (Invitrogen, Carlsbad, CA, USA) according to the manufacturers' protocol. Precipitations of exosomes were fully lysed by Trizol LS (Invitrogen Life Technologies, Paisley, UK) for subsequent RNA extraction. Total RNAs were extracted using miRNeasy mini kit (Qiagen, Venlo, Netherlands) based on the manufacturers' protocol. The purity and concentration of isolated RNAs were determined from OD260/280 readings by use of a spectrophotometer. cDNA were synthesized using a PrimeScriptTM RT reagent Kit (Takara, Tianjin, China) according to the manufacturers' instructions, and stored at −20°C for further analysis.

### Microarray analysis for differentially expressed miRNAs

Exosomes from serum samples of 3 primary CRC patients and 3 healthy controls were selected for microarray analysis (GenechemCo., Ltd., Shanghai, China) for differentially expressed miRNAs in serum. The quality of total RNAs from serum exosomes was evaluated by an Agilent 2100 Bioanalyzer (Agilent Technologies, Santa Clara, CA, USA). Total RNAs were labeled using Flashtag^TM^ Biotin HSR RNA Labeling kit for Affymetrix GeneChip miRNA Arrays (Genisphere, Hatfield, Pennsylvania, USA) following the manufacturer's instructions, and then competitively hybridised to a miRNA array (Affymetrix miRNA Expression Microarray analysis) based on the manufacturer's protocol. The hybridisation signal was scanned and by GeneChip Scanner 3000.

### qRT-PCR for the validation of differentially expressed miRNAs

qRT-PCR was carried out in triplicate assay in accordance with the specifications of miRNA qRT-PCR SYBR^®^ Kit (Takara, Tianjin, China) to validate the differentially expressed miRNAs in serum exosomes. The amplification protocol consisted of an the denaturation step at 95°C for 10 min, followed by 40 cycles of 95°C for 10 s and 60°C for 60 s.

### Statistical analysis

Data were presented as mean ± SEM. Independent-Samples *T* test or One-Way ANOVA was used for statistical analysis. Softwares of SPSS (version 16.0) and Graphpad (version 5.0) were used for calculation. *P* < 0.05 was considered to be statistically significant.
